# Quantitative Analysis of Differential Proteome Expression in Bladder Cancer vs. Normal Bladder Cells Using SILAC Method

**DOI:** 10.1371/journal.pone.0134727

**Published:** 2015-07-31

**Authors:** Ganglong Yang, Zhipeng Xu, Wei Lu, Xiang Li, Chengwen Sun, Jia Guo, Peng Xue, Feng Guan

**Affiliations:** 1 The Key Laboratory of Carbohydrate Chemistry & Biotechnology, Ministry of Education, School of Biotechnology, Jiangnan University, Wuxi, China; 2 Shaanxi Provincial People’s Hospital, Xi’an, China; 3 Wuxi Medical School, Jiangnan University, Wuxi, China; 4 Department of Urology, Affiliated Hospital of Jiangnan University, Wuxi, China; 5 Laboratory of Proteomics, Institute of Biophysics, Chinese Academy of Sciences, Beijing, China; Pacific Northwest National Laboratory, UNITED STATES

## Abstract

The best way to increase patient survival rate is to identify patients who are likely to progress to muscle-invasive or metastatic disease upfront and treat them more aggressively. The human cell lines HCV29 (normal bladder epithelia), KK47 (low grade nonmuscle invasive bladder cancer, NMIBC), and YTS1 (metastatic bladder cancer) have been widely used in studies of molecular mechanisms and cell signaling during bladder cancer (BC) progression. However, little attention has been paid to global quantitative proteome analysis of these three cell lines. We labeled HCV29, KK47, and YTS1 cells by the SILAC method using three stable isotopes each of arginine and lysine. Labeled proteins were analyzed by 2D ultrahigh-resolution liquid chromatography LTQ Orbitrap mass spectrometry. Among 3721 unique identified and annotated proteins in KK47 and YTS1 cells, 36 were significantly upregulated and 74 were significantly downregulated with >95% confidence. Differential expression of these proteins was confirmed by western blotting, quantitative RT-PCR, and cell staining with specific antibodies. Gene ontology (GO) term and pathway analysis indicated that the differentially regulated proteins were involved in DNA replication and molecular transport, cell growth and proliferation, cellular movement, immune cell trafficking, and cell death and survival. These proteins and the advanced proteome techniques described here will be useful for further elucidation of molecular mechanisms in BC and other types of cancer.

## Introduction

Bladder cancer (BC) is the fifth most common type of human cancer. There were an estimated 74,690 newly diagnosed cases and 15,580 deaths from this disease in the United States in 2013 [1]. Of total BC patients, >70% have nonmuscle-invasive disease and ~25% present initially with muscle invasion. Patients with the muscle-invasive form have a 50% risk of distant metastases and a poor prognosis [2]. The recurrence of superficial bladder tumors is a major reason for the worldwide prevalence of BC [3]. The majority (90%) of BCs are classified histologically as urothelial carcinomas (UCs), derived from the bladder urothelium [4]. Bladder epithelial tissues have a clear hierarchical organization consisting of three morphologically distinct cell types: basal, intermediate, and umbrella cells, corresponding respectively to early, middle, and late differentiation states [5]. Malignant transformation may occur in each of these cell types, resulting in a diversity of tumor phenotypes [6]. According to the latest report of the American Cancer Society, the relative 5-year survival rate for BC with early detection (stage I, (T1, N0, M0)) is ~88% [7]. Therefore, identification of novel early-stage molecular markers is desirable for improved risk stratification.

Candidate biomarkers for BC detection evaluated to date include telomerase, bladder tumor antigen (BTA), nuclear matrix protein 22 (NMP-22), and fibrin degradation product (FDP). The reliability of tests based on these biomarkers is poor because of low sensitivity and high false-positive rates [8–11]. Proteins can potentially be identified specific to aggressive or nonaggressive types of cancer. Proteome analysis is challenging because of the limited amount of available clinical sample [12]. Monitoring of the proteome of BC cells could provide additional information for clinical diagnostic purposes.

Recent advances in mass spectrometry (MS) for protein identification and quantification facilitate in-depth analysis of large numbers of proteins, and have been used for examination of the whole proteome in several systems. Such methods include 2D difference gel electrophoresis (2D DIGE), the similar iTRAQ (isobaric tag for relative and absolute quantitation), isotope-coded affinity tagging (ICAT), and stable isotope labeling by amino acids in cell culture (SILAC) [13–15]. In comparison with peptide-based absolute quantitation methods, SILAC has the advantages of mixing samples at the very beginning, and reduced sample-to-sample variability. Metabolic labeling with stable isotopes has been described as the "gold standard" in protein quantification [16]. Arginine (Arg) and lysine (Lys) are the stable isotope-labeled amino acids most frequently used in SILAC-based studies, because subsequent trypsin digestion of isolated proteins (which cleaves at basic residues) for MS analysis generates peptides with a single labeled amino acid, simplifying analysis and quantification [17]. In the present study, three stable isotopes each of Arg (R0, R6, R10) and Lys (K0, K4, K8)in three separate cultures (“light” (L), “medium” (M), and “heavy” (H)) were used to analyze proteome differences at various stages of BC. Distinctive L, M, and H forms of each peptide as detected by MS reflected relative amounts of the corresponding protein in three isotopically encoded BC cell stages.

**Fig 1:**
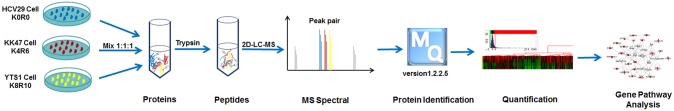
Schematic procedure for quantitative analysis of proteins in BC cells vs. normal bladder cells.

Three human cell lines were studied: normal bladder epithelial HCV29, low grade nonmuscle invasive bladder cancer (NMIBC) KK47, and metastatic muscle invasive bladder cancer YTS1. Each of the three cell lines was cultured in media added with three combinations of unlabeled Lys and Arg ("light"), D_4_-Lys and ^13^C_6_-Arg ("medium"), and ^13^C_6_^15^N_2_-Lys and ^13^C_6_^15^N_4_-Arg ("heavy"). Proteins with >98% label incorporation were analyzed and quantified by 2D-HPLC-LTQ Orbitrap MS (Fig 1). Differential expression of the identified proteins, which are presumably related to BC development, was confirmed by western blotting, quantitative RT-PCR, and cell staining with specific antibodies.

## Material and Methods

### Cell culture

HCV29, KK47, and YTS1 cells were established as described previously [18–20] and kindly donated by Dr. Sen-itiroh Hakomori (The Biomembrane Institute; Seattle, WA, USA). Cells were cultured in RPMI 1640 medium supplemented with 10% FBS and 1% penicillin/ streptomycin at 37°C in 5% CO_2_ atmosphere. For SILAC labeling, cells were cultured in SILAC-labeled RPMI 1640 with 10% dialyzed FBS and 1% penicillin/ streptomycin containing “light”(K0R0), “medium”(K4R6), or “heavy”(K8R10) Lys and Arg. To prevent Arg-to-Pro conversion, L-Pro (200 mg/L) was added to the medium as described previously [21]. Cells were cultured for at least 5 passages to eliminate nonlabeled Lys and Arg.

### Cell lysis and protein extraction

Total proteins of the three cell lines were lysed and extracted using T-PER Reagent (Thermo Scientific; San Jose, CA, USA) according to the manufacturer's instruction. In brief, cells (~1×10^7^) were detached with trypsin, washed twice with ice-cold 1×PBS (0.01 M phosphate buffer containing 0.15 M NaCl, pH 7.4), lysed with 1 mL T-PER reagent containing protease inhibitors (1 mM PMSF and 0.1% aprotinin), incubated for 30 min on ice, homogenized, and centrifuged at 12,000 rpm for 15 min. The supernatant was harvested and stored at -80°C. Protein concentration was determined by BCA assay (Beyotime; Haimen, China).

### SDS-PAGE and *in-gel* digestion

Proteins were separated by 10% SDS-PAGE, visualized by Coomassie staining for 2 hr, and destained overnight. Excised gel slices were washed with 25 mM ammonium bicarbonate/ 50% acetonitrile (ACN). Dried pieces were incubated with 20 μL of 10 mM dithiothreitol (DTT) at 56°C for 1 hr and then with an equal volume of 20 mM iodoacetamide (IAM) at room temperature in the dark. Gel slices were washed and trypsinized with 20 μL (10 ng/μL) trypsin (Promega; Madison, WI, USA) for 30 min at 4°C. Excess trypsin solution was removed, 20 μL of 25 mM NH_4_HCO_3_ was added, and the mixture was incubated overnight at 37°C. Extracted products were dried with a SpeedVac concentrator (CentriVap Cold Trap, Labconco; Kansas, MO, USA) [22].

### MALDI-TOF/TOF-MS

Dried samples were dissolved with 0.1% trifluoroacetic acid (TFA), spotted onto an MTP AnchorChip sample target, and air-dried. Peptides were recrystallized with matrix α-cyanohydroxycinnamic acid (CHCA; 1 μL of 10 mg/mL) and characterized by MALDI-TOF/TOF-MS (UltrafleXtreme, Bruker Daltonics; Bremen, Germany). Ionization was achieved by irradiation with a nitrogen laser (λ = 337 nm) operating at 20 Hz. Mass spectra were acquired using the FlexControl and FlexAnalysis software programs.

### In-solution digestion

Proteins from three types of stable isotope-labeled cells were mixed at 1:1:1, reduced, and alkylated by incubation with equal amounts of 10 mM DTT and 20 mM IAM. Alkylated proteins were digested by trypsin added at a ratio of 1:50 (w/w) and incubated overnight at 37°C [23]. Total peptides were concentrated and desalted using a 10KD size-exclusion spin ultrafiltration unit and dried using a SpeedVac concentrator.

### LC-MS/MS analysis

2D-LC-MS was performed using LTQ Orbitrap MS (Thermo Fisher Scientific; Waltham, MA, USA) as described previously [24]. Digested peptides (100 μg) were injected into a biphasic capillary column (i.d. 200 μm) packed with C_18_ resin (ReproSil-Pur, 5 μm, Dr. Maisch GmbH) and strong cation-exchange resin (Luna 5 μm SCX 100A, Phenomenex). Peptide effluents from the biphasic column at each step were directed into a 15-cm C_18_ analytical column (i.d. 75 μm, ReproSil-Pur, 3 μm) at flow rate 500 nl/min. Nano-ESI was performed with spray voltage 2.0 kV and heated capillary temperature 200°C. One full MS scan (300–1800) in the Orbitrap was followed by five MS/MS scans of the five most intense ions selected from the MS spectrum in LTQ. Charge state screening was enabled for +2, +3, +4, and above [25].

### Data analysis

Raw MS data were analyzed using the MaxQuant software program (V. 1.2.2.5) [26,27]. A false discovery rate (FDR) of 0.01 for proteins and peptides and a minimum peptide length of 6 amino acids were required. MS/MS spectra were searched by Andromeda [28] against the IPI human database (V. 3.85). The MaxQuant program determined the SILAC state of peptides from mass differences between SILAC peptide pairs, and this information was used to perform searches with fixed Arg6 and Lys4 or Arg10 and Lys8 modifications, as appropriate. Quantification in MaxQuant was performed as described previously [26].

Differential regulation within each experimental M/L ("medium/ light") ratio and H/L ("heavy/ light") ratio of the identified proteins was normalized using z-score analysis, as described previously [29,30]. In brief, M/L and H/L ratios were converted into log2 space, and average ratios and SD (standard deviations) were calculated for each data set. The log2 M/L and H/L ratio of each protein were converted into a z-score, using the following formula:

$$\text{z}‐\text{scores}\left(\sigma \right)\text{ of }\left[\text{b}\right]\;=\;\frac{{\text{log}}_{2}\frac{X\left(M\;or\;H\right)}{L}\left[b\right]-Average\;of\;\left({\text{log}}_{2}of\;each\;number,\;a\ldots n\right)}{Standard\;deviation\;of\;\left({\text{log}}_{2}of\;each\;number,\;a\ldots n\right)}$$

where b were deemed as a single protein in a data set population (a….n). The z-score was a measure of how many SD units (σ) of the log2 M/L or H/L ratio of the protein was away from the population mean. A z-score ≥1.960σ represented that differential expression of the protein lied outside the 95% confidence interval, a score ≥2.576σ represented expression outside the 99% confidence interval, and a score ≥3.291σ represented 99.9% confidence. Z-scores ≥1.960σ were considered to be significant [29].

### Functional annotation and Ingenuity Pathways Analysis

Identified proteins were further analyzed using the SWISS-PROT database to classify their biological process, cellular component, and molecular function [31]. Significant over-represented gene ontology (GO) terms were identified using the Database for Annotation, Visualization and Integrated Discovery (DAVID) gene bioinformatic resources [32, 33]. Proteins determined to be differentially regulated as described in the preceding section were tabulated in Excel and their International Protein Index (IPI) numbers were uploaded into DAVID (http://david.abcc.ncifcrf.gov/home.jsp) for functional annotation analysis. Data sets containing gene identifiers and corresponding expression values were then uploaded into the Ingenuity Systems application. Each IPI number was mapped to its corresponding gene object in the Ingenuity Pathways Knowledge Base. Networks of the proteins were generated algorithmically based on their connectivity. Fisher's exact test was used to calculate a p-value indicating the probability that a particular biological function and/or disease assigned to that network was due to chance alone.

### Western blotting

Western blotting was performed as described previously [34]. In brief, proteins were separated on 10% SDS-PAGE gel, transferred onto a PVDF membrane, and the membrane was blocked using 5% nonfat milk in TBST and probed using specific antibodies. The primary antibodies were rabbit anti-insulin-like growth factor 2 mRNA-binding protein 1 (IGF2BP1) (#AP10466b, Abgent; San Diego, CA, USA), rabbit anti-melanoma-associated antigen 4 (MAGEA4) (#AP6166a, Abgent), rabbit anti-Thy-1 membrane glycoprotein (THY1) (#AP2050a, Abgent), 14-3-3 protein sigma (SFN) (#sc-365539, Santa Cruz Biotechnology, Dallas, TX, USA), rabbit anti-fibronectin (FN1) (#F3648, Sigma-Aldrich), mouse anti-vimentin (VIM) (#V5255, Sigma-Aldrich), CD70 antigen (CD70) (#sc-7681, Santa Cruz Biotechnology), and mouse anti-β-catenin (CTNNB1) (#610153, BD Biosciences; San Jose, CA, USA). The secondary antibodies were appropriate horseradish peroxidase (HRP)-conjugated rabbit anti-mouse or goat anti-rabbit (#A0216 and #A0208, Beyotime). Bands were visualized using an enhanced chemiluminescence detection kit (Westar Nova, Cyanagen; Bologna, Italy).

### Quantitative real-time PCR

Cells (1×10^5^ per well in a 6-well plate) were cultured and treated as described above. Total RNA was isolated using an RNApure Tissue Kit (CWBiotech; Beijing, China) according to the manufacturer’s instructions. Primers were designed using the DNAMAN program (V. 6.0.3; Lynnon Biosoft, Canada). First-strand cDNA was synthesized from total RNA using ReverTra Ace-α (Toyobo; Osaka, Japan). Quantitative real-time PCR was performed by LightCycler-based SYBR Green I dye detection with UltraSYBR Mixture (CWBiotech). Gene expression was quantified by the 2^-ΔΔCT^ method [35].

### Cell staining

Cells were cultured on sterilized coverslips in 24-well plates until 70–80% confluence, washed, immobilized, permeabilized with 0.2% Triton X-100 for 10 min at room temperature, and blocked with 5% nonfat milk overnight at 4°C. Fixed cells were incubated with diluted primary antibodies for 12 hr, incubated with FITC-labeled secondary antibodies at 4°C for 6 hr in the dark, washed, stained with 4 μg/mL DAPI at room temp for 10 min, washed with 1×PBS, and photographed with a fluorescence microscope (Eclipse E600, Nikon; Tokyo, Japan).

## Results

### Determination of isotope incorporation efficiency

**Fig 2:**
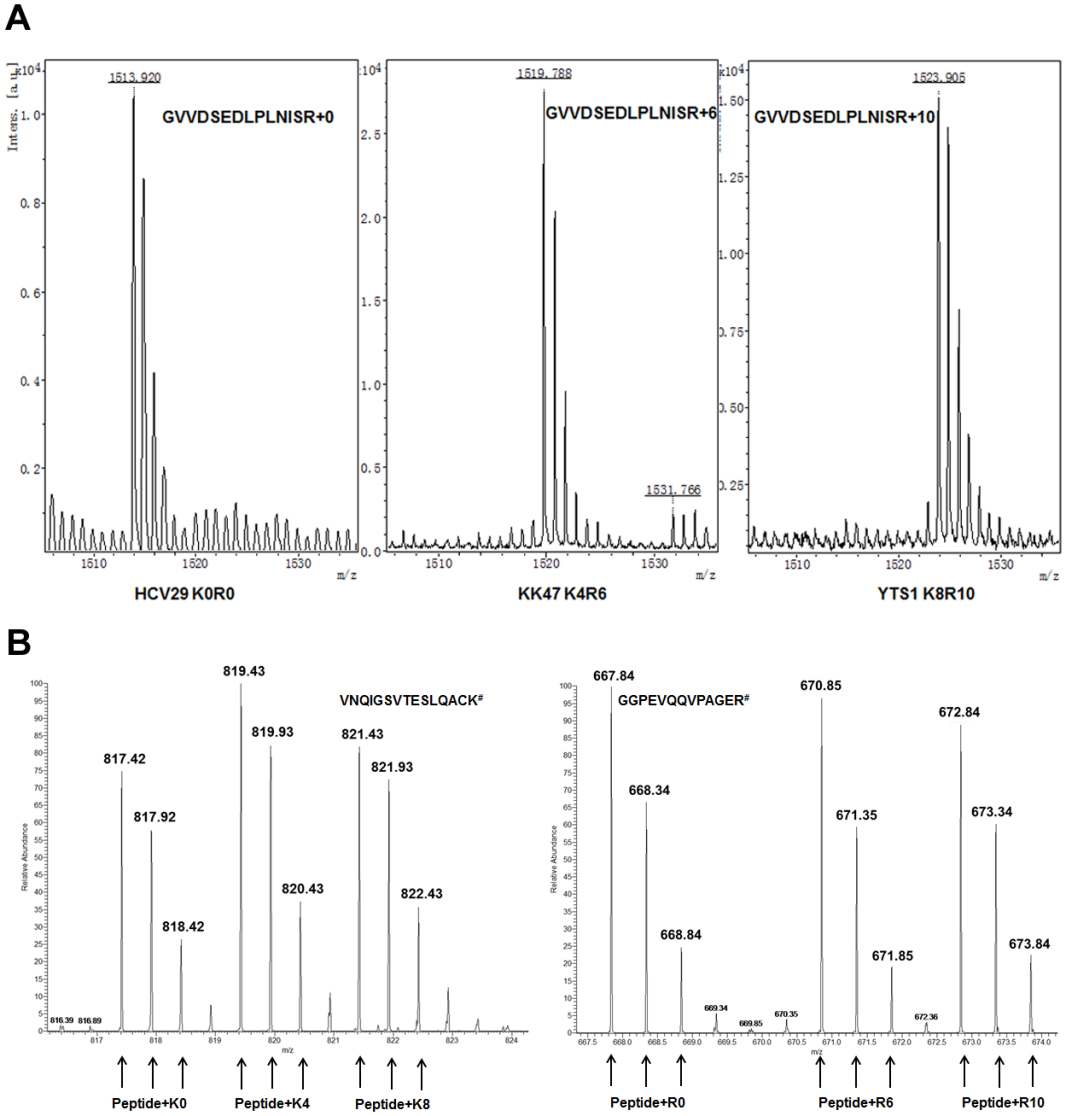
Mass spectrometric analysis of stable isotope-labeled proteins (SILAC method). (A) Determination of incorporation efficiency by MALDI-TOF/TOF-MS. Peaks annotated as R0 (left), R6 (middle), and R10 (right) are peptide GVVDSEDLPLNISR of heat shock protein 90 from HCV29, KK47, and YTS1 cells. (B) Identification and quantification of proteome in BC cells by 2D-HPLC LTQ Orbitrap MS. Peaks annotated as K0, K4, and K8 (left) and R0, R6, and R10 (right) are doubly charged peptide VNQIGSVTESLQACK of alpha enolase and GGPEVQQVPAGER of fatty acid synthase.

To analyze dynamic changes in BC oncogenesis at the proteome level, the SILAC method was applied to three bladder cell lines to obtain labeled cell populations. Sufficient labeling is a prerequisite for reliable quantification using this method. In the case of incomplete labeling of proteins, particularly for labeling efficiency <95%, quantitation of low-abundance proteins would be masked by contaminated “light” peptides. To determine incorporation efficiency of labeled Lys and Arg, “light” (HCV29), “medium” (KK47), and “heavy” (YTS1) proteins were separated, and the high-abundance protein was *in-gel* digested. MALDI-TOF/TOF-MS results for peptide GVVDSEDLPLNISR in heat shock protein 90 (P08238) indicated that complete incorporation of isotopically labeled Arg and Lys was achieved in KK47 and YTS1, and no Arg-to-Pro conversion occurred (Fig 2A). LC-ESI-MS/MS analysis of the doubly charged peptide VNQIGSVTESLQACK of alpha-enolase (P06733) and GGPEVQQVPAGER of fatty acid synthase (P49327) showed that these doublets of actual peak clusters were from HCV29, KK47, and YTS1 cells, respectively (Fig 2B).

### SILAC cell model for quantification of proteome in BC progression

**Fig 3:**
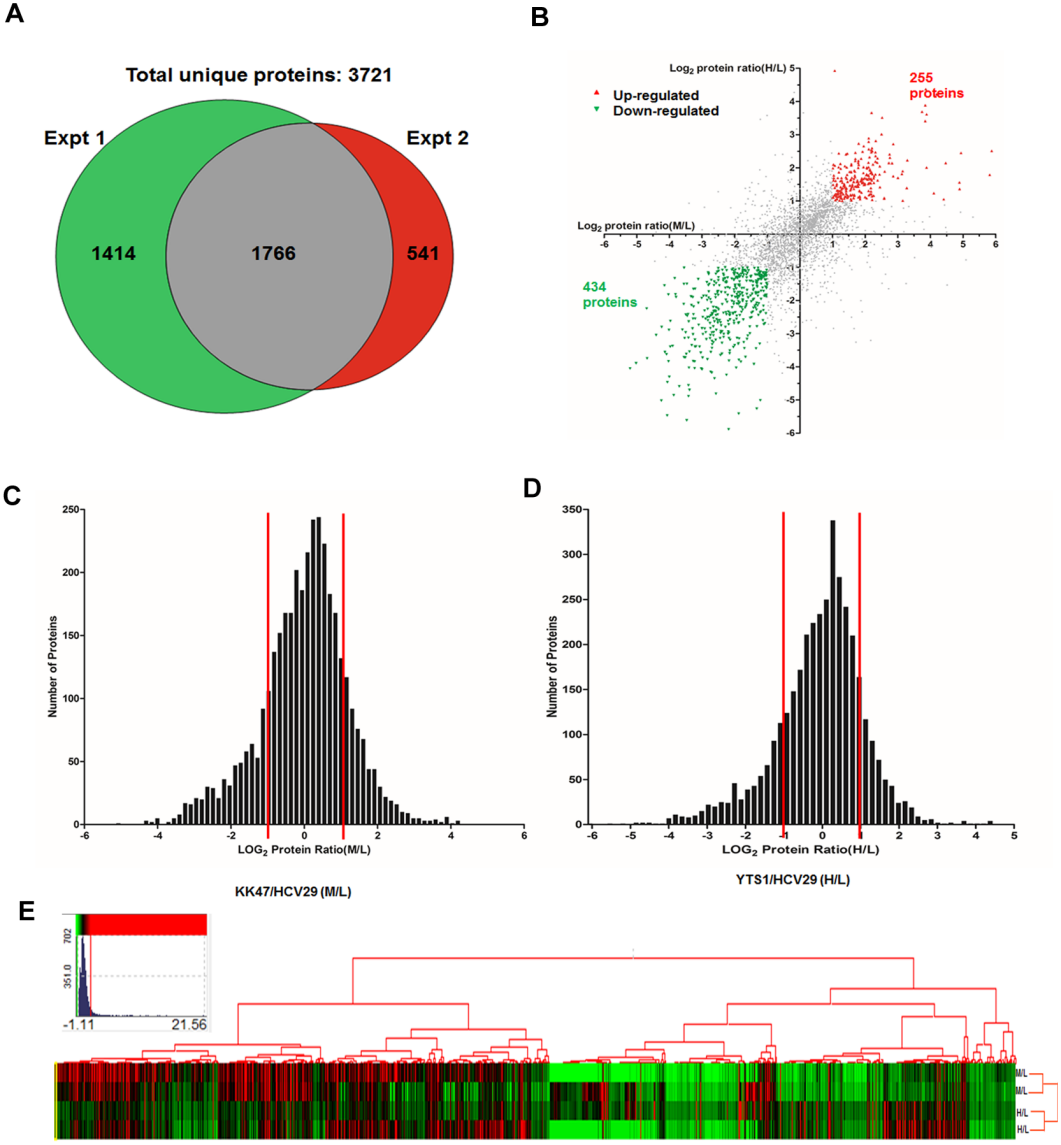
Distributions of proteins identified in various experiments described in the text. (A) Venn diagrams of numbers of identified proteins from individual experiments. (B) Ratios of KK47/HCV29 (M/L) and YTS1/HCV29 (H/L) for the set of 1766 proteins. log2 of the SILAC ratio for each protein (n = 2) reflects differences in relative expression among KK47, YTS1, and HCV29 cells. (C) Distribution of SILAC M/L ratios. (D) Distribution of SILAC H/L ratios. (E) Cluster graph ("heat map") generated by hierarchical clustering of significant regulated proteins after averaging z-scores using a 95% cutoff.

Proteins isolated from the three cell lines were mixed (1:1:1) and digested using a 10 KD filter (Millipore; Billerica, MA, USA). Peptides were analyzed by ultrahigh-resolution liquid chromatography-tandem MS (nLC-ESI-MS/MS) on a hybrid linear ion trap LTQ Orbitrap instrument. A total of 3721 unique proteins were identified in two independent replicate experiments (Fig 3A and S1 Table). Of these, 1766 proteins (47.5% of the total) that were identified in both experiments and satisfied the criteria established for protein quantitation were subjected to further bioinformatic analysis. The distribution histograms of log ratios for both M/L and H/L fit a Gaussian distribution. Most of the identified proteins were within the ±1 range of log ratios (Fig 3C and 3D). Using 1 as the threshold log ratio, expression of 255 proteins was higher in both KK47 and HCV29, and expression of 434 proteins was lower in both KK47 and HCV29 (Fig 3B). Population distribution-based z-scores allowed direct comparison of proteins from different experiments. Differing confidence level cutoffs were applied to the data by z-score analysis to determine which proteins were significantly differentially regulated. The cutoffs applied were 95%, 99% and 99.9%, corresponding to z-scores of ±1.960, ±2.576, and ±3.291, respectively. Using a 95% cutoff, significant differential regulation was observed for 110 proteins in KK47 vs. HCV29 (36 upregulated, 74 downregulated) and for 87 proteins in YTS1 vs. HCV29 (17 up, 70 down). Differential regulation was observed for 35 proteins in the two BC lines vs. HCV29 using a 99% cutoff (2 up, 33 down), but for only five of these proteins using a 99.9% cutoff (2 up, 3 down) (Table 1). Tables 2 and 3 list the upregulated and downregulated proteins determined in the two experiments, with their average SILAC ratios and z-scores. All proteins differentially regulated with >95% confidence had a >5-fold alteration of SILAC ratios, and most proteins differentially regulated with >99% confidence had a >10-fold alteration of SILAC ratios.

**Table 1:** Protein number, log2 ratio mean±SD, and z-scores of SILAC-labeled proteins.

Cell line	Mean log 2	SD Log 2	z-scores^a^		
			±1.960σ	±2.576σ	±3.291σ
**KK47 (M/L)**	-0.072	1.237	36, 74	13, 22	7, 3
**YTS1 (H/L)**	-0.151	1.143	17, 70	6, 27	1, 3
**Both cell lines**			2, 33	2, 3	0, 0

^a^The first and second value shown are, respectively, the number of upregulated and downregulated proteins outside the indicated confidence level.

**Table 2:** Upregulated proteins in BC cells with >95% confidence^a^.

Swiss-prot	Gene name	Protein name	M/L average	H/L average	log2 M/L average	log2 H/L average	z-scores M/L	z-scores H/L
**C9JGI3_HUMAN**	TYMP	Thymidine phosphorylase	39.51	1.43	5.30	0.51	4.35	0.47
**FOLR1_HUMAN**	FOLR1	Folate receptor alpha	31.73	2.91	4.99	1.54	4.09	1.30
**J3QRJ3_HUMAN**	THY1	Thy-1 membrane glycoprotein	29.72	2.57	4.89	1.36	4.01	1.16
**RCN3_HUMAN**	RCN3	Reticulocalbin-3	23.97	3.29	4.58	1.72	3.76	1.45
**STEA4_HUMAN**	STEAP4	Metalloreductase STEAP4	23.43	4.23	4.55	2.08	3.74	1.74
**EF1A2_HUMAN**	EEF1A2	Elongation factor 1-alpha 2	17.58	0.87	4.14	-0.20	3.40	-0.10
**1433S_HUMAN**	SFN	14-3-3 protein sigma	16.87	3.27	4.08	1.71	3.35	1.44
**DDX21_HUMAN**	DDX21	Nucleolar RNA helicase 2	14.33	10.65	3.84	3.41	3.16	2.82
**KCRB_HUMAN**	CKB	Creatine kinase B-type	14.15	1.30	3.82	0.38	3.15	0.37
**CPSM_HUMAN**	CPS1	Carbamoyl-phosphate synthase [ammonia], mitochondrial	12.22	0.99	3.61	-0.01	2.98	0.05
**MTAP_HUMAN**	MTAP	S-methyl-5-thioadenosine phosphorylase	11.66	10.09	3.54	3.33	2.92	2.75
**ASSY_HUMAN**	ASS1	Argininosuccinate synthase	11.08	1.86	3.47	0.89	2.86	0.78
**H0YDA6_HUMAN**	NAPRT1	Nicotinate phosphoribosyltransferase	10.23	2.82	3.35	1.49	2.77	1.27
**1A68_HUMAN**	HLA-A	HLA class I histocompatibility antigen, A-32 alpha chain	8.34	0.44	3.06	-1.19	2.53	-0.91
**EPIPL_HUMAN**	EPPK1	Epiplakin	8.12	0.86	3.02	-0.21	2.50	-0.11
**PGH2_HUMAN**	PTGS2	Prostaglandin G/H synthase 2	7.95	1.13	2.99	0.17	2.48	0.20
**HPDL_HUMAN**	HPDL	4-hydroxyphenylpyruvate dioxygenase-like protein	7.93	1.13	2.99	0.18	2.47	0.20
**K1C18_HUMAN**	KRT18	Keratin, type I cytoskeletal 18	7.51	2.18	2.91	1.13	2.41	0.97
**WDR3_HUMAN**	WDR3	WD repeat-containing protein 3	7.26	3.36	2.86	1.75	2.37	1.47
**PADI2_HUMAN**	PADI2	Protein-Arg deiminase type-2	6.99	1.38	2.81	0.46	2.33	0.43
**EXOS4_HUMAN**	EXOSC4	Exosome complex component RRP41	6.57	3.20	2.72	1.68	2.26	1.42
**A6PVX1_HUMAN**	SELENBP1	Selenium-binding protein 1	6.41	1.04	2.68	0.06	2.23	0.10
**KYNU_HUMAN**	KYNU	Kynureninase	6.32	0.35	2.66	-1.50	2.21	-1.16
**4F2_HUMAN**	SLC3A2	4F2 cell-surface antigen heavy chain	6.24	0.88	2.64	-0.18	2.19	-0.09
**PO210_HUMAN**	NUP210	Nuclear pore membrane glycoprotein 210	6.16	1.05	2.62	0.07	2.18	0.12
**G3V588_HUMAN**	ITPK1	Inositol-tetrakisphosphate 1-kinase	6.09	1.71	2.61	0.77	2.17	0.68
**ANM7_HUMAN**	PRMT7	Protein Arg N-methyltransferase 7	5.99	2.18	2.58	1.13	2.15	0.97
**S100P_HUMAN**	S100P	Protein S100-P	5.92	0.62	2.57	-0.69	2.13	-0.50
**B0S8I7_HUMAN**	LAGE3	L antigen family member 3	5.87	4.50	2.55	2.17	2.12	1.81
**J3QKT4_HUMAN**	PYCR1	Pyrroline-5-carboxylate reductase;	5.72	2.35	2.52	1.23	2.09	1.05
**MCMBP_HUMAN**	MCMBP	Mini-chromosome maintenance complex-binding protein	5.65	4.77	2.50	2.25	2.08	1.88
**PRI2_HUMAN**	PRIM2	DNA primase large subunit	5.47	4.59	2.45	2.20	2.04	1.84
**K1C17_HUMAN**	KRT17	Keratin, type I cytoskeletal 17	5.39	0.89	2.43	-0.16	2.02	-0.07
**LAT1_HUMAN**	SLC7A5	Large neutral amino acids transporter small subunit 1	5.35	1.27	2.42	0.34	2.01	0.34
**MYPN_HUMAN**	MYPN	Myopalladin	5.34	0.88	2.42	-0.19	2.01	-0.09
**NPM3_HUMAN**	NPM3	Nucleoplasmin-3	5.29	3.29	2.40	1.72	2.00	1.45
**UHRF1_HUMAN**	UHRF1	E3 ubiquitin-protein ligase UHRF1	4.98	5.13	2.32	2.36	1.93	1.97
**ITA6_HUMAN**	ITGA6	Integrin alpha-6	4.58	12.65	2.19	3.66	1.83	3.02
**CND3_HUMAN**	NCAPG	Condensin complex subunit 3	4.04	5.67	2.01	2.50	1.69	2.08
**UBS3B_HUMAN**	UBASH3B	Ubiquitin-associated and SH3 domain-containing protein B	3.88	6.30	1.96	2.66	1.64	2.21
**E9PD53_HUMAN**	SMC4	Structural maintenance of chromosomes protein	3.51	5.72	1.81	2.52	1.52	2.09
**KIF4A_HUMAN**	KIF4A	Chromosome-associated kinesin KIF4A	3.26	6.56	1.70	2.71	1.44	2.25
**MRP_HUMAN**	MARCKSL1	MARCKS-related protein	2.74	5.39	1.45	2.43	1.23	2.02
**CTRO_HUMAN**	CIT	Citron Rho-interacting kinase	2.66	5.31	1.41	2.41	1.20	2.01
**CIP2A_HUMAN**	KIAA1524	Protein CIP2A	1.87	5.15	0.90	2.36	0.79	1.97
**MAGA4_HUMAN**	MAGEA4	Melanoma-associated antigen 4	1.48	17.47	0.56	4.13	0.51	3.40
**PLEK2_HUMAN**	PLEK2	Pleckstrin-2	0.98	5.95	-0.02	2.57	0.04	2.14
**AL1A3_HUMAN**	ALDH1A3	Aldehyde dehydrogenase family 1 member A3	0.93	13.75	-0.11	3.78	-0.03	3.12
**RGS10_HUMAN**	RGS10	Regulator of G-protein signaling 10	0.79	6.50	-0.34	2.70	-0.22	2.24
**E41L3_HUMAN**	EPB41L3	Band 4.1-like protein 3	0.72	6.54	-0.47	2.71	-0.33	2.25
**UCHL1_HUMAN**	UCHL1	Ubiquitin carboxyl-terminal hydrolase isozyme L1	0.42	9.45	-1.25	3.24	-0.95	2.68

^a^Proteins shown have at least one z-score value (M/L or H/L) ≥1.960σ in two biological replicates.

**Table 3:** Downregulated proteins in BC cells with >95% confidence^a^.

Swiss-prot	Gene name	Protein name	M/L average	H/L average	log2 M/L average	log2 H/L average	z-scores M/L	z-scores H/L
**1C07_HUMAN**	HLA-C	HLA class I histocompatibility antigen	0.17	0.02	-2.53	-6.05	-1.99	-4.83
**A8MUB1_HUMAN**	TUBA4A	Tubulin alpha-4A chain	0.35	0.09	-1.51	-3.42	-1.16	-2.70
**AL1B1_HUMAN**	ALDH1B1	Aldehyde dehydrogenase X, mitochondrial	0.11	0.10	-3.14	-3.34	-2.48	-2.64
**ALDR_HUMAN**	AKR1B1	Aldose reductase	0.11	0.04	-3.13	-4.58	-2.48	-3.65
**ANO10_HUMAN**	ANO10	Anoctamin-10	0.16	0.17	-2.65	-2.53	-2.09	-1.99
**ARMC9_HUMAN**	ARMC9	LisH domain-containing protein ARMC9	0.16	0.12	-2.67	-3.10	-2.10	-2.45
**ASC_HUMAN**	PYCARD	Apoptosis-associated speck-like protein containing a CARD	0.15	0.38	-2.72	-1.39	-2.14	-1.06
**AT2B4_HUMAN**	ATP2B4	Plasma membrane calcium-transporting ATPase 4	0.16	0.06	-2.65	-3.96	-2.08	-3.15
**B2MG_HUMAN**	B2M	Beta-2-microglobulin	0.42	0.10	-1.24	-3.29	-0.95	-2.60
**BCAT1_HUMAN**	BCAT1	Branched-chain-amino-acid aminotransferase, cytosolic	0.09	0.57	-3.41	-0.80	-2.70	-0.59
**BIN1_HUMAN**	BIN1	Myc box-dependent-interacting protein 1	0.21	0.12	-2.22	-3.09	-1.74	-2.44
**CATB_HUMAN**	CTSB	Cathepsin B	0.07	0.37	-3.84	-1.45	-3.05	-1.11
**CAV1_HUMAN**	CAV1	Caveolin-1;Caveolin	0.16	0.38	-2.68	-1.40	-2.11	-1.08
**CBPA4_HUMAN**	CPA4	Carboxypeptidase A4	0.11	0.08	-3.18	-3.73	-2.51	-2.96
**CD70_HUMAN**	CD70	CD70 antigen	0.16	0.10	-2.65	-3.26	-2.09	-2.58
**CD97_HUMAN**	CD97	CD97 antigen	1.98	0.17	0.99	-2.59	0.86	-2.04
**CD99_HUMAN**	CD99	CD99 antigen	0.14	0.82	-2.84	-0.28	-2.24	-0.17
**CKAP4_HUMAN**	CKAP4	Cytoskeleton-associated protein 4	0.16	0.23	-2.64	-2.12	-2.07	-1.66
**CNN3_HUMAN**	CNN3	Calponin-3	0.11	0.42	-3.25	-1.25	-2.57	-0.95
**CO3_HUMAN**	C3	Complement C3	0.84	0.15	-0.26	-2.77	-0.15	-2.18
**CO6A2_HUMAN**	COL6A2	Collagen alpha-2(VI) chain	0.07	0.26	-3.81	-1.96	-3.02	-1.52
**CO6A3_HUMAN**	COL6A3	Collagen alpha-3(VI) chain	0.12	0.17	-3.11	-2.56	-2.46	-2.01
**CO7A1_HUMAN**	COL7A1	Collagen alpha-1(VII) chain	0.34	0.10	-1.56	-3.39	-1.20	-2.68
**COPZ2_HUMAN**	COPZ2	Coatomer subunit zeta-2	0.06	0.02	-4.07	-5.47	-3.23	-4.37
**CPPED_HUMAN**	CPPED1	Calcineurin-like phosphoesterase domain-containing protein 1	0.10	0.11	-3.33	-3.15	-2.63	-2.49
**D6RJ89_HUMAN**	ACOX3	Peroxisomal acyl-coenzyme A oxidase 3	0.18	0.12	-2.44	-3.07	-1.92	-2.43
**DCBD2_HUMAN**	DCBLD2	Discoidin, CUB and LCCL domain-containing protein 2	0.29	0.16	-1.77	-2.68	-1.38	-2.11
**DOP2_HUMAN**	DOPEY2	Protein dopey-2	0.06	0.28	-4.03	-1.82	-3.20	-1.42
**DPYL3_HUMAN**	DPYSL3	Dihydropyrimidinase-related protein 3	0.10	0.40	-3.27	-1.31	-2.59	-1.00
**E7EUD0_HUMAN**	DKK3	Dickkopf-related protein 3	0.17	0.20	-2.57	-2.34	-2.02	-1.83
**ES8L2_HUMAN**	EPS8L2	Epidermal growth factor receptor kinase substrate 8-like protein 2	0.34	0.09	-1.56	-3.47	-1.20	-2.75
**F5GY03_HUMAN**	SPARC	SPARC	0.12	0.15	-3.01	-2.76	-2.38	-2.17
**F8WCU2_HUMAN**	FKBP7	Peptidyl-prolyl cis-trans isomerase	0.16	0.19	-2.67	-2.38	-2.10	-1.87
**FA49A_HUMAN**	FAM49A	Protein FAM49A	0.13	0.14	-2.96	-2.83	-2.34	-2.23
**FHL1_HUMAN**	FHL1	Four and a half LIM domains protein 1	0.13	0.09	-2.89	-3.55	-2.28	-2.81
**FHL2_HUMAN**	FHL2	Four and a half LIM domains protein 2	0.14	0.17	-2.88	-2.58	-2.27	-2.03
**FINC_HUMAN**	FN1	Fibronectin	0.11	0.13	-3.25	-2.96	-2.57	-2.33
**FKB10_HUMAN**	FKBP10	Peptidyl-prolyl cis-trans isomerase FKBP10	0.67	0.07	-0.57	-3.80	-0.40	-3.01
**FLNC_HUMAN**	FLNC	Filamin-C	0.17	2.65	-2.59	1.41	-2.03	1.19
**FPRP_HUMAN**	PTGFRN	Prostaglandin F2 receptor negative regulator	0.25	0.06	-1.99	-4.09	-1.55	-3.25
**FUCO_HUMAN**	FUCA1	Tissue alpha-L-fucosidase	0.14	0.16	-2.82	-2.60	-2.22	-2.05
**G3V2M6_HUMAN**	STAT2	Signal transducer and activator of transcription 2	0.19	0.17	-2.41	-2.52	-1.89	-1.98
**GABT_HUMAN**	ABAT	4-aminobutyrate aminotransferase, mitochondrial	0.03	0.15	-5.07	-2.72	-4.04	-2.14
**GBP1_HUMAN**	GBP1	Interferon-induced guanylate-binding protein 1	0.12	0.13	-3.09	-2.93	-2.44	-2.31
**GBP2_HUMAN**	GBP2	Interferon-induced guanylate-binding protein 2	0.09	0.12	-3.46	-3.09	-2.74	-2.44
**GELS_HUMAN**	GSN	Gelsolin	0.11	0.25	-3.14	-1.99	-2.48	-1.55
**GLSK_HUMAN**	GLS	Glutaminase kidney isoform, mitochondrial	0.17	0.37	-2.53	-1.43	-1.99	-1.10
**GPX1_HUMAN**	GPX1	Glutathione peroxidase 1	0.74	0.11	-0.44	-3.14	-0.30	-2.48
**H0Y8D1_HUMAN**	PRSS1	Trypsin-1	0.05	0.06	-4.22	-4.01	-3.36	-3.18
**H0YGX7_HUMAN**	ARHGDIB	Rho GDP-dissociation inhibitor 2	0.09	1.91	-3.40	0.93	-2.69	0.81
**H7C2T5_HUMAN**	POFUT2	GDP-fucose protein O-fucosyltransferase 2	0.13	0.31	-2.91	-1.67	-2.30	-1.29
**H7C5L1_HUMAN**	PTGES2	Prostaglandin E synthase 2	1.46	0.12	0.55	-3.11	0.50	-2.46
**HM13_HUMAN**	HM13	Minor histocompatibility antigen H13	0.17	0.26	-2.52	-1.95	-1.98	-1.52
**HYEP_HUMAN**	EPHX1	Epoxide hydrolase 1	0.26	0.13	-1.93	-2.96	-1.50	-2.34
**ICAM1_HUMAN**	ICAM1	Intercellular adhesion molecule 1	0.58	0.13	-0.80	-2.91	-0.58	-2.30
**ITA3_HUMAN**	ITGA3	Integrin alpha-3	0.13	0.19	-2.90	-2.39	-2.29	-1.87
**ITAV_HUMAN**	ITGAV	Integrin alpha-V	0.12	0.13	-3.05	-2.90	-2.41	-2.29
**ITPR3_HUMAN**	ITPR3	Inositol 1,4,5-trisphosphate receptor type 3	0.30	0.11	-1.76	-3.15	-1.36	-2.49
**JAM1_HUMAN**	F11R	Junctional adhesion molecule A	0.06	0.08	-4.09	-3.63	-3.25	-2.87
**K1C10_HUMAN**	KRT10	Keratin, type I cytoskeletal 10	0.13	0.12	-2.95	-3.06	-2.33	-2.41
**K1C9_HUMAN**	KRT9	Keratin, type I cytoskeletal 9	0.13	0.09	-2.89	-3.49	-2.28	-2.76
**K2C1_HUMAN**	KRT1	Keratin, type II cytoskeletal 1	0.12	0.11	-3.07	-3.12	-2.42	-2.47
**K7ENN8_HUMAN**	TRIM16	Tripartite motif-containing protein 16	0.21	0.11	-2.22	-3.21	-1.74	-2.54
**L1CAM_HUMAN**	L1CAM	Neural cell adhesion molecule L1	0.43	0.10	-1.22	-3.33	-0.92	-2.63
**LAMB1_HUMAN**	LAMB1	Laminin subunit beta-1	0.16	1.20	-2.64	0.27	-2.08	0.27
**LASP1_HUMAN**	LASP1	LIM and SH3 domain protein 1	0.16	0.18	-2.69	-2.44	-2.12	-1.91
**LEG3_HUMAN**	LGALS3	Galectin-3	0.71	0.09	-0.49	-3.55	-0.34	-2.81
**LRP1_HUMAN**	LRP1	Prolow-density lipoprotein receptor-related protein 1	0.20	0.12	-2.33	-3.01	-1.83	-2.38
**MAOX_HUMAN**	ME1	NADP-dependent malic enzyme;Malic enzyme	0.31	0.11	-1.67	-3.23	-1.29	-2.55
**MAP1B_HUMAN**	MAP1B	Microtubule-associated protein 1B	0.09	0.24	-3.44	-2.09	-2.72	-1.63
**MICA2_HUMAN**	MICAL2	Protein-methionine sulfoxide oxidase MICAL2	0.18	0.26	-2.51	-1.95	-1.97	-1.52
**ML12A_HUMAN**	MYL12A	Myosin regulatory light chain 12A	0.09	0.19	-3.43	-2.41	-2.71	-1.89
**MMP14_HUMAN**	MMP14	Matrix metalloproteinase-14	0.10	0.27	-3.34	-1.88	-2.64	-1.46
**MOT4_HUMAN**	SLC16A3	Monocarboxylate transporter 4	0.72	0.06	-0.47	-4.00	-0.32	-3.17
**MYH9_HUMAN**	MYH9	Myosin-9	0.13	0.22	-2.97	-2.17	-2.34	-1.70
**MYL9_HUMAN**	MYL9	Myosin regulatory light polypeptide 9	0.11	0.16	-3.19	-2.67	-2.52	-2.10
**NIBAN_HUMAN**	FAM129A	Protein Niban	0.13	1.19	-2.94	0.25	-2.32	0.26
**NMES1_HUMAN**	NMES1	Normal mucosa of esophagus-specific gene 1 protein	0.40	0.06	-1.33	-3.95	-1.02	-3.14
**NXP20_HUMAN**	FAM114A1	Protein NOXP20	0.23	0.15	-2.14	-2.73	-1.68	-2.15
**OAS3_HUMAN**	OAS3	2-5-oligoadenylate synthase 3	0.25	0.16	-2.02	-2.63	-1.58	-2.07
**PAI2_HUMAN**	SERPINB2	Plasminogen activator inhibitor 2	0.08	0.42	-3.64	-1.25	-2.88	-0.95
**PDLI1_HUMAN**	PDLIM1	PDZ and LIM domain protein 1	0.15	0.39	-2.76	-1.35	-2.18	-1.03
**PEA15_HUMAN**	PEA15	Astrocytic phosphoprotein PEA-15	0.15	0.37	-2.74	-1.43	-2.16	-1.10
**PLSL_HUMAN**	LCP1	Plastin-2	0.11	0.06	-3.20	-3.95	-2.53	-3.13
**PML_HUMAN**	PML	Protein PML	0.14	0.15	-2.79	-2.70	-2.20	-2.13
**PNKD_HUMAN**	PNKD	Probable hydrolase PNKD	0.18	0.14	-2.48	-2.84	-1.95	-2.24
**PPGB_HUMAN**	CTSA	Lysosomal protective protein	0.17	0.28	-2.58	-1.84	-2.03	-1.43
**PTRF_HUMAN**	PTRF	Polymerase I and transcript release factor	0.15	0.53	-2.74	-0.91	-2.16	-0.68
**Q5RHS7_HUMAN**	S100A2	Protein S100-A2	1.27	0.17	0.35	-2.59	0.34	-2.04
**RAI14_HUMAN**	RAI14	Ankycorbin	0.14	0.43	-2.87	-1.21	-2.26	-0.92
**RCN1_HUMAN**	RCN1	Reticulocalbin-1	0.46	0.16	-1.11	-2.67	-0.84	-2.10
**RGPS2_HUMAN**	RALGPS2	Ras-specific guanine nucleotide-releasing factor RalGPS2	0.10	0.40	-3.32	-1.32	-2.63	-1.01
**S10A6_HUMAN**	S100A6	Protein S100-A6	0.31	0.07	-1.71	-3.91	-1.32	-3.10
**SAMH1_HUMAN**	SAMHD1	SAM domain and HD domain-containing protein 1	0.35	0.11	-1.53	-3.21	-1.18	-2.54
**SELM_HUMAN**	SELM	Selenoprotein M	0.72	0.14	-0.48	-2.81	-0.33	-2.21
**SERPH_HUMAN**	SERPINH1	Serpin H1	0.44	0.17	-1.18	-2.57	-0.89	-2.02
**SH3B4_HUMAN**	SH3BP4	SH3 domain-binding protein 4	0.11	0.08	-3.15	-3.68	-2.49	-2.92
**SNX18_HUMAN**	SNX18	Sorting nexin-18	0.05	1.04	-4.41	0.06	-3.51	0.11
**SNX3_HUMAN**	SNX3	Sorting nexin-3	0.09	0.27	-3.48	-1.91	-2.76	-1.49
**STC2_HUMAN**	STC2	Stanniocalcin-2	0.24	0.17	-2.04	-2.54	-1.59	-2.00
**SYCM_HUMAN**	CARS2	Probable cysteine—tRNA ligase, mitochondrial	0.25	0.14	-1.98	-2.82	-1.54	-2.22
**TGM2_HUMAN**	TGM2	Protein-glutamine gamma-glutamyltransferase 2	0.32	0.07	-1.66	-3.79	-1.29	-3.01
**TPM1_HUMAN**	TPM1	Tropomyosin alpha-1 chain	0.06	0.15	-4.05	-2.74	-3.21	-2.16
**UAP1L_HUMAN**	UAP1L1	UDP-N-acetylhexosamine pyrophosphorylase-like protein 1	0.15	0.19	-2.76	-2.42	-2.17	-1.90
**UBA6_HUMAN**	UBA6	Ubiquitin-like modifier-activating enzyme 6	0.17	0.22	-2.58	-2.20	-2.03	-1.72
**UBA7_HUMAN**	UBA7	Ubiquitin-like modifier-activating enzyme 7	0.15	0.08	-2.72	-3.66	-2.14	-2.90
**UN13D_HUMAN**	UNC13D	Protein unc-13 homolog D	0.39	0.10	-1.38	-3.36	-1.05	-2.66
**VAT1_HUMAN**	VAT1	Synaptic vesicle membrane protein VAT-1 homolog	0.16	0.21	-2.63	-2.25	-2.07	-1.76
**VIME_HUMAN**	VIM	Vimentin	0.15	0.38	-2.75	-1.40	-2.17	-1.07
**WDFY1_HUMAN**	WDFY1	WD repeat and FYVE domain-containing protein 1	0.08	0.30	-3.60	-1.72	-2.85	-1.33
**WIPI1_HUMAN**	WIPI1	WD repeat domain phosphoinositide-interacting protein 1	0.18	0.13	-2.50	-2.97	-1.96	-2.35

^a^Proteins shown have at least one z-score value (M/L or H/L) ≥1.960σ in two biological replicates.

Hierarchical clustering analysis of samples was performed to examine correlations of proteome patterns among the three cell lines. The Cluster graph ("heat map") shows that samples of the same cell line cluster together (Fig 3E). Some proteomes were distinctive among the three cell lines based on significant alterations in metastatic vs. low grade nonmuscle invasive, whereas other proteomes were moderate and consistent. Proteins in the former group may be involved in BC development.

### Functional classification and pathway analysis of identified proteins

**Fig 4:**
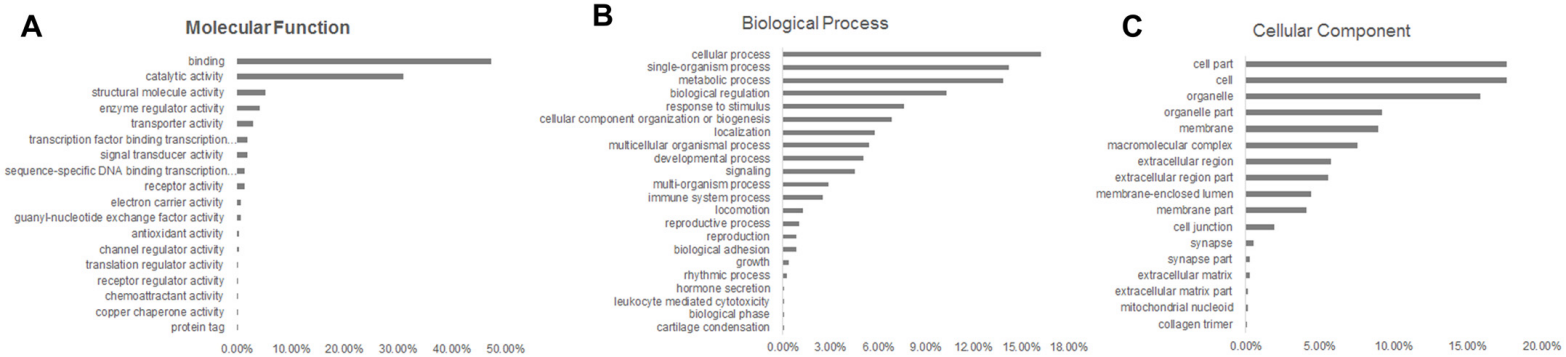
Functional classification of identified proteins using SWISS-PROT database based on universal GO annotation terms. Proteins shown were linked to at least one annotation term within the GO molecular function (A), biological process (B), and cellular component (C) categories.

Functional interpretation is a crucial step in data analysis when extensive functional annotation of the data sets is not available. Taking into account their nonexclusive localization in GO, the identified proteins were linked to at least one annotation term each within the GO molecular function, biological process, and molecular component categories. The most common molecular functions were binding (47.2%), and catalytic activity (30.9%) (Fig 4A). The major biological process categories were cellular (16.3%), single-organism (14.2%), and metabolic (13.8%) (Fig 4B). The major cellular component categories were cell (17.6%), cell part (17.6%), and organelle (15.8%) (Fig 4C).

To identify enrichment terms associated with the upregulated and downregulated groups of proteins after averaging of z-scores using the 95% cutoff, lists of proteins were uploaded to the DAVID website using the complete human proteome as background. To help clarify which molecular functions and biological processes were most affected during BC maturation, over-represented GO terms were identified based on threshold count ≥ 2 and Expression Analysis Systematic Explorer (EASE) < 0.1. The over-represented molecular functions, biological processes, and cellular components in the significant enriched GO terms of upregulated proteins were analyzed. The most highly ranked molecular function was neutral amino acid transmembrane transporter activity (2 proteins). The most highly ranked biological processes were cellular amino acid derivative metabolic process (4 proteins), ribonucleoprotein complex biogenesis (4 proteins), response to extracellular stimulus (4 proteins), and nitrogen compound biosynthetic process (4 proteins). The most highly ranked cellular components were intracellular non-membrane-bounded organelle (14 proteins) and non-membrane-bounded organelle (14 proteins).

Next, over-represented molecular functions, biological processes, and cellular components in the significant enriched GO terms of downregulated proteins were analyzed. The most highly ranked molecular functions were calcium ion binding (16 proteins), structural molecule activity (11 proteins), and identical protein binding (10 proteins). The most highly ranked biological processes were cell adhesion (14 proteins), biological adhesion (14 proteins), response to wounding (13 proteins), and immune response (13 proteins). The most highly ranked cellular components were plasma membrane (40 proteins), plasma membrane part (30 proteins), and non-membrane-bounded organelle (24 proteins) (S2 Table).

**Fig 5:**
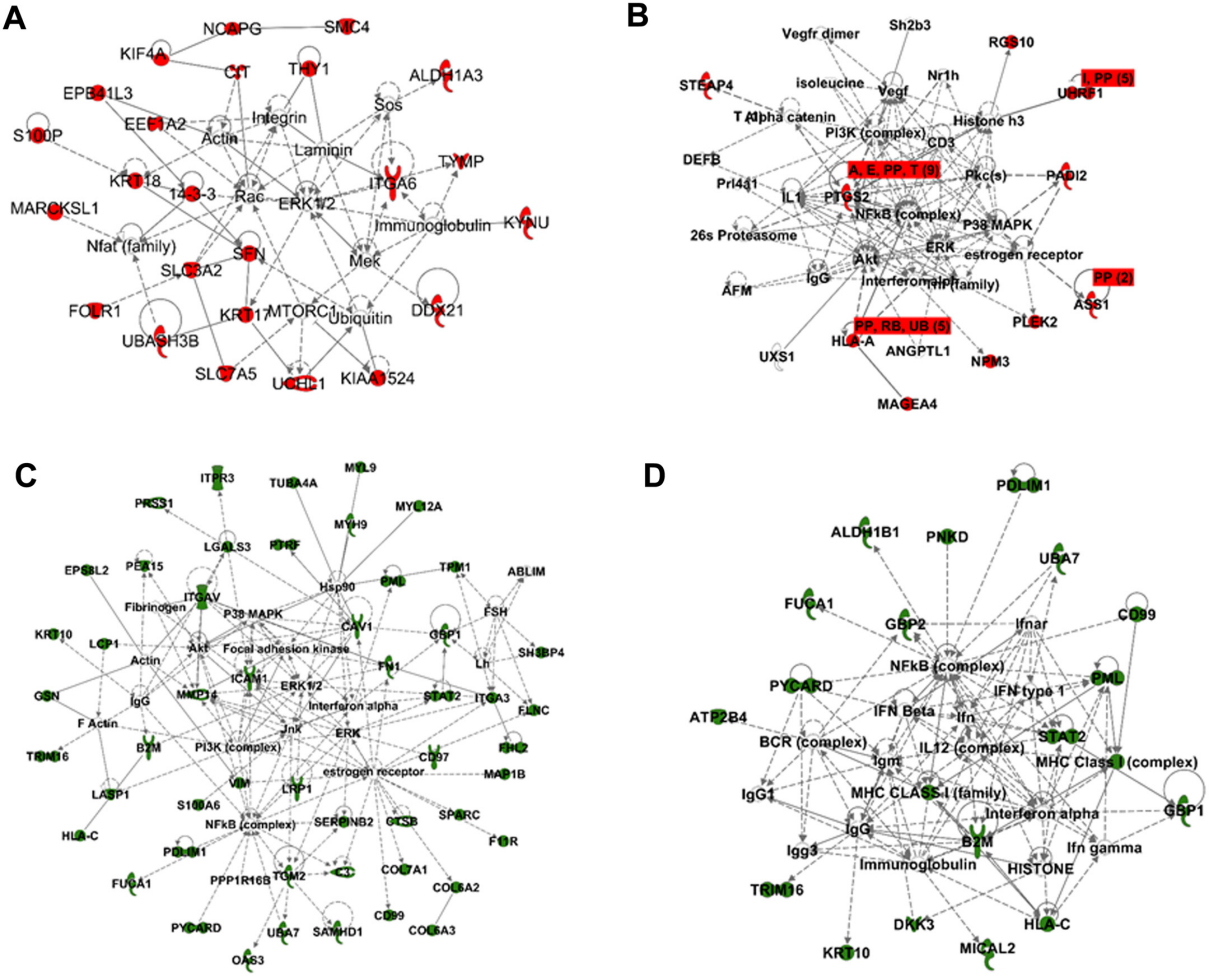
Functional network analysis of differentially regulated proteins with z-score cutoff 95% observed in different stages of BC cells using Ingenuity Pathways Analysis (IPA). (A and B) Top network functions of DNA replication, molecular transport, cell growth, and cell proliferation for upregulated proteins. (C and D) Top network functions of cellular movement, immune cell trafficking, and lipid metabolism. Solid lines: direct known interactions. Dashed lines: suspected or indirect interactions. White: proteins known to be in the network but not identified in our study.

Proteins were further analyzed, and metabolic and canonical pathways and interconnecting proteins were generated, using Ingenuity Pathways Analysis (IngenuityH Systems, www.ingenuity.com). The top network functions identified as upregulated proteins in BC cells were involved in DNA replication, amino acid metabolism, molecular transport (52 proteins; Fig 5A), gene expression and hereditary disorders (33 proteins), cell growth and proliferation (20 proteins; Fig 5B), and post-translational modification and cancer (4 proteins). The top network functions identified as downregulated proteins in BC cells were cellular movement and immune cell trafficking (97 proteins; Fig 5C), lipid metabolism (31 proteins; Fig 5D), cellular development, growth and proliferation, and cell death and survival (6 proteins). These findings indicate that BC cell proteomes were continuously shifting depending on the stage of cell metastasis.

### Confirmation of MS results by western blotting

**Fig 6:**
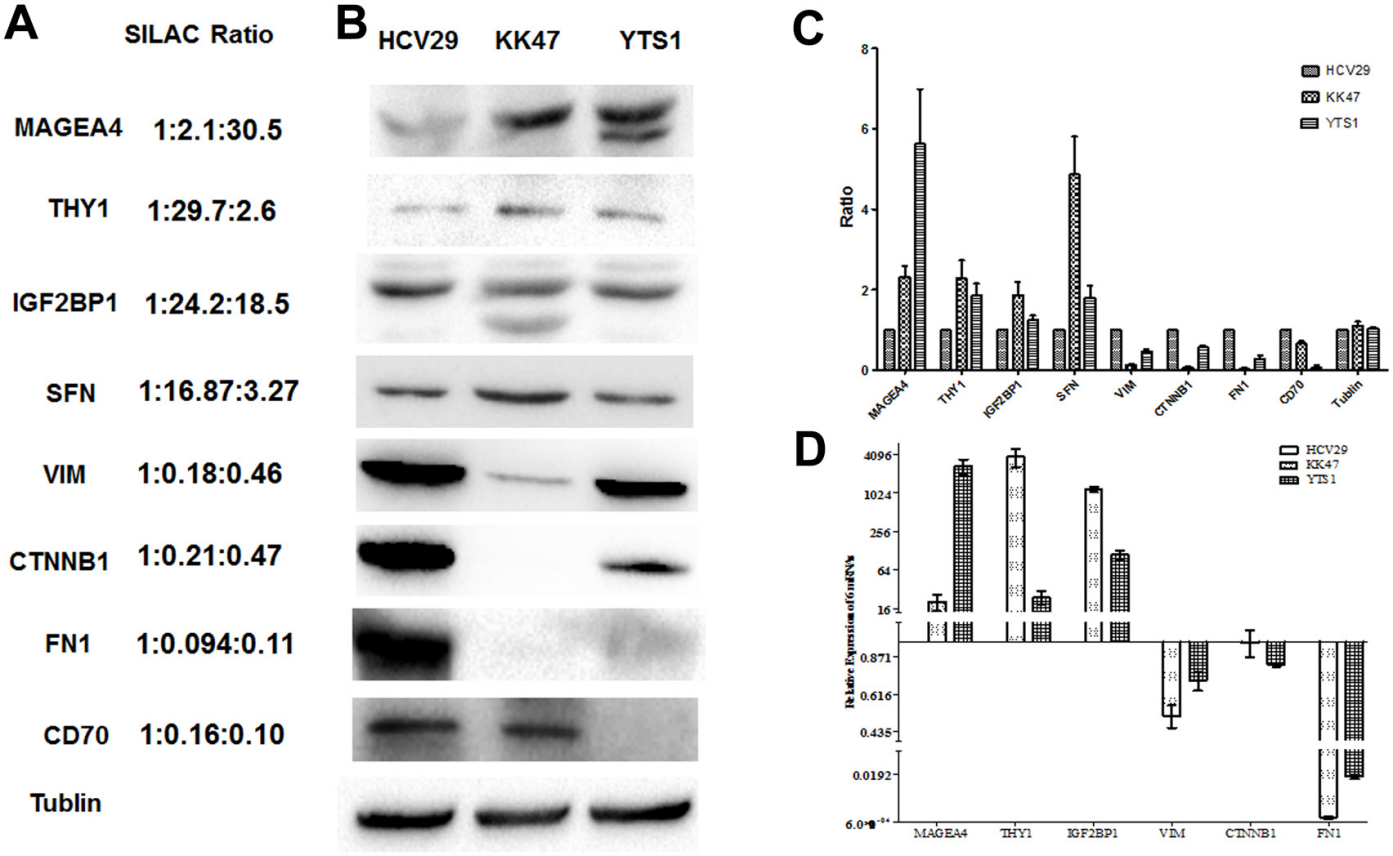
Confirmation of SILAC-determined protein and RNA abundances. (A) SILAC L:M:H average ratios for six selected proteins. (B) Western blotting analysis of selected proteins. Proteins were transferred to a PVDF membrane, probed with their primary antibodies, and incubated with HRP-conjugated rabbit anti-mouse or goat anti-rabbit secondary antibodies. (C) Densitometric quantitation of the protein levels. The protein was normalized by the tublin, and then compared to HCV29, which were arbitrarily set at 1.0. (D) Gene expression for the proteins was analyzed by qRT-PCR. Relative expression in comparison to control samples was analyzed by the 2^−ΔΔCt^ method and represented as Log_2_. Expression of genes above Log_2_(2) or below Log_2_(1/2) was significantly upregulated or downregulated, respectively.

Variations of the differential proteins described above were confirmed by western blotting. THY1, MAGEA4, IGF2BP1 and SFN were detected at higher levels in BC KK47 and YTS1 cells than in normal bladder epithelial HCV29 cells, whereas VIM, CTNNB1, FN1 and CD70 were detected at lower levels in KK47 and YTS1 than in HCV29 (Fig 6B and 6C). In general, the western blotting results were consistent with the variables from MS analysis (Fig 6A; S1 Table and S1 Fig).

### Confirmation of SILAC results by qRT-PCR

The expression of six responding genes at the transcriptional level was evaluated by qRT-PCR. In BC KK47 and YTS1 cells, expression of *MAGEA4*, *THY1*, and *IGF2BP1* was significantly increased, whereas that of *VIM*, *CTNNB1*, and *FN1* was greatly reduced (Fig 6D). These findings are consistent with SILAC results.

### Confirmation of SILAC and western blotting results by cell staining with antibodies

**Fig 7:**
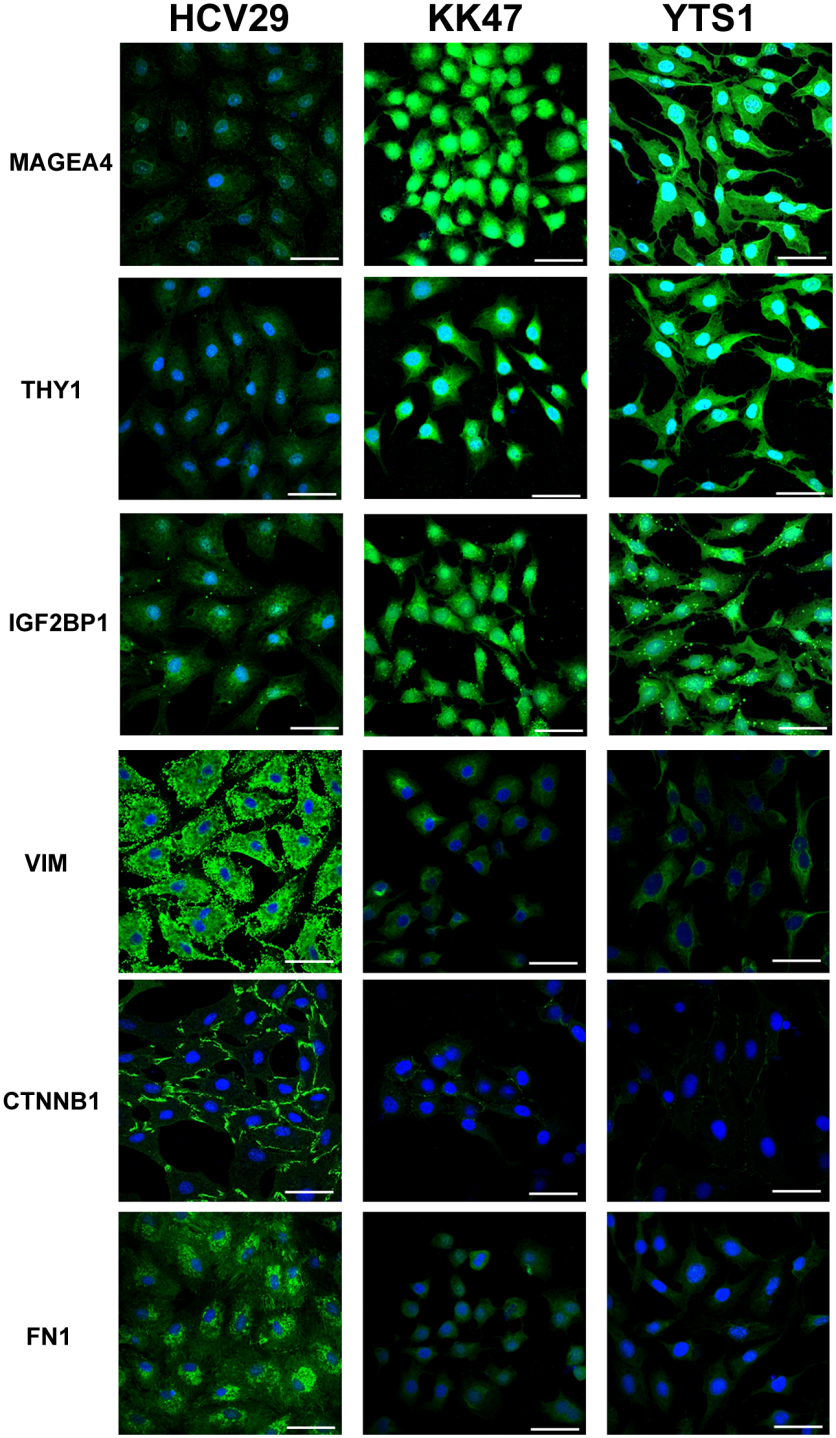
Differential expression revealed by cell staining. HCV29, KK47, and YTS1 cells were cultured and stained with six antibodies directed to identified proteins (MAGEA4, THY1, IGF2BP1, VIM, CTNNB1, FN1) labeled with Cy3 as described in M&M. Images are shown of merge images of Cy3-conjugated antibodies and DAPI staining of nuclei (objective magnification 60×). Scale bars: 70 μm.

Antibodies recognizing MAGEA4, THY1, IGF2BP1, VIM, CTNNB1, and FN1 proteins, whose expression differed significantly in BC cells vs. HCV29 cells, were used to confirm the results of previous analyses and to assess protein distributions. Fluorescence signal intensities in the two BC cell lines were significantly higher for MAGEA4, THY1, and IGF2BP1 and significantly lower for VIM, CTNNB1, and FN1, in agreement with SILAC, western blotting, and RT-PCR results. Preferential localization was observed for MAGEA4 in central cytoplasm (including mitochondria and centrosome) and the nuclear region, for THY1 and VIM in cytoplasmic membrane and cytoplasm, for IGF2BP1 in the nuclear region and cytoplasm, and for CTNNB1 and FN1 in cytoplasmic membrane (Fig 7).

## Discussion

Urothelial carcinoma of the bladder is unique among epithelial carcinomas in its divergent pathways of tumorigenesis. At the time of diagnosis for transitional cell BC, ~80% of patients are in the low-grade (grade 1–2) non-muscle invasive (cTa or cT1) stage. In patients with low grade Ta disease, the 15-year progression-free survival is 95% with no cancer-specific mortality [36]. Therefore, biomarkers are needed for predicting a risk of stage progression from non-invasive to invasive, or non-metastatic to metastatic and predicting responsiveness to systemic therapies. The human cell lines HCV29 (normal bladder epithelia), KK47 (low grade nonmuscle invasive bladder cancer), and YTS1 (metastatic bladder cancer) have been widely used in studies of molecular mechanisms and cell signaling during the progression of bladder cancer to muscle or metastatic states [37]. However, little attention has been paid to global quantitative proteome analysis of these three cell lines.

Quantitative analysis of proteins at different stages of BC progression is a challenging but important task for understanding of disease mechanisms. SILAC, a differential isotope labeling strategy that involves metabolic labeling of proteins *in vivo*, has been widely applied in cell biology and studies of model organisms such as yeast, bacteria, nematodes, plants, and mice [38, 39]. In SILAC, natural “light” isotopes of carbon, nitrogen, and hydrogen in amino acids incorporated during protein translation are substituted with "heavy" isotopes such as ^13^C, ^15^N, and ^2^H. No study to date has quantified differences in protein abundance at various stages of BC. Previous studies have focused on comparative protein levels in BC patients vs. control subjects, but not on alterations of protein levels during BC progression [8,9].

Identification and characterization of protein levels at various steps of differentiation are essential for our understanding of normal tissue development and malignant transformation. In the present study, we applied the SILAC method to analysis of HCV29, KK47, and YTS1. This strategy provided protein information for all three cell lines during one MS experiment. Following normalization by the z-score method, differential regulation was observed for 110 proteins in KK47 vs. HCV29 and for 87 proteins in YTS1 vs. HCV29. These differentially regulated proteins, which may play important roles in BC development, include SFN (14-3-3 protein sigma), SELENBP1 (selenium-binding protein 1), COL6A3 (collagen α3 (VI) chain), and CD70. COL6A2 and COL6A3 protein levels are reduced in urine of BC patients [40]. SFN is downregulated in invasive bladder transitional cell carcinomas undergoing epithelial-to-mesenchymal conversion and highly upregulated in pure squamous cell carcinomas [41]. Some of the proteins that are altered in BC are also altered in other cancers. In particular, expression of FOLR1 (folate receptor alpha) is increased relative to normal tissue in many types of epithelial cancer, including non-mucinous ovarian, endometrial, non-small cell lung, colorectal, and breast cancers [42].

The DAVID bioinformatic resources provide a set of powerful tools to explore their large gene lists in depth from many different biological perspectives in order to fully extract associated biological meanings. Enrichment analysis of GO terms in our identified proteins indicated that neutral amino acid transmembrane transporter activity and metabolic processes were significantly upregulated and that protein binding, cell adhesion, biological adhesion, and immune response were significantly downregulated in the BC cell lines. The observed changes in GO terms tended to promote cellular proliferation, tumor development, cancer cell progression and metastasis, and escape from immune system surveillance.

A major challenge in cancer biology is the formulation of biological hypotheses regarding biomarker candidates [43]. Pathway analysis of differentially expressed proteins indicates that DNA replication and molecular transport, cell growth, and cell proliferation are requirements for cancer cell metastasis. Intercellular adhesion molecule-1 (iCAM-1) is a transmembrane glycoprotein present at basal levels in a wide variety of cell types and is upregulated in response to a number of inflammatory mediators [44]. The biological significance of iCAM-1 expression in cancer remains controversial; it is elevated in gastric, breast, oral, and thyroid cancer tissues [44–47] but reduced in some ovarian adenocarcinoma cell lines and primary tumors [48]. Also, iCAM-1 expression is up-regulated in squamous cell types associated with inflammation, such as schistosomal bladder cancer [49]. However, in the present study, iCAM-1 expression was reduced in metastatic bladder cancer cells. Moreover, CIT, a novel tissue-specific Ser/Thr kinase that encompasses the Rho-Rac-binding protein Citron, plays a role in cytokinesis and in Rho signaling that modulates myosin phosphorylation and cell adhesion [50]. Upregulation of CIT promotes DNA replication and cancer cell proliferation.

In conclusion, we successfully applied the SILAC method to identify and quantify proteins whose level is significantly up- or downregulated during BC development or progression. Similar advanced proteome techniques will be useful for further elucidation of biomarkers and molecular mechanisms in BC and other types of cancer.

## Supporting Information

S1 Fig Protein raw ion intensities for western blot. (TIF)

S1 Table The full list of proteins identified by 2D-LC-MS/MS. (XLSX)

S2 Table Gene ontology statistics of significantly upregulated and downregulated proteins. (XLSX)
