# Supplementary figures and images for: Quantitative Analysis of Differential Proteome Expression in Bladder Cancer vs. Normal Bladder Cells Using SILAC Method
Source: PLoS One. 2015 Jul 31;10(7):e0134727. doi: 10.1371/journal.pone.0134727 (PMC4521931; doi:10.1371/journal.pone.0134727)

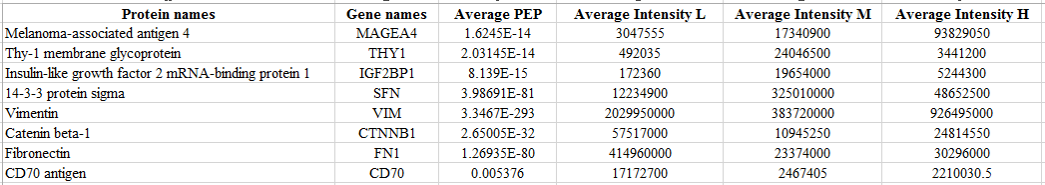

Supplement: S1 Fig — (TIF) [file pone.0134727.s001.tif]
